# Progress Toward Polio Eradication — Worldwide, January 2018–March 2020

**DOI:** 10.15585/mmwr.mm6925a4

**Published:** 2020-06-26

**Authors:** Anna N. Chard, S. Deblina Datta, Graham Tallis, Cara C. Burns, Steven G.F. Wassilak, John F. Vertefeuille, Michel Zaffran

**Affiliations:** ^1^Epidemic Intelligence Service, CDC; ^2^Global Immunization Division, Center for Global Health, CDC; ^3^Polio Eradication Department, World Health Organization, Geneva, Switzerland; ^4^Division of Viral Diseases, National Center for Immunization and Respiratory Diseases, CDC.

Since the Global Polio Eradication Initiative (GPEI) was established in 1988, two of the three wild poliovirus (WPV) serotypes (types 2 and 3) have been eradicated.* Transmission of WPV type 1 (WPV1) remains uninterrupted only in Afghanistan and Pakistan. This report summarizes progress toward global polio eradication during January 1, 2018–March 31, 2020 and updates previous reports (*1*,*2*). In 2019, Afghanistan and Pakistan reported the highest number of WPV1 cases (176) since 2014. During January 1–March 31, 2020 (as of June 19), 54 WPV1 cases were reported, an approximate fourfold increase from 12 cases during the corresponding period in 2019. Paralytic poliomyelitis can also be caused by circulating vaccine-derived poliovirus (cVDPV), which emerges when attenuated oral poliovirus vaccine (OPV) virus reverts to neurovirulence following prolonged circulation in underimmunized populations (*3*). Since the global withdrawal of type 2-containing OPV (OPV2) in April 2016, cVDPV type 2 (cVDPV2) outbreaks have increased in number and geographic extent (*4*). During January 2018–March 2020, 21 countries reported 547 cVDPV2 cases. Complicating increased poliovirus transmission during 2020, the coronavirus disease 2019 (COVID-19) pandemic and mitigation efforts have resulted in suspension of immunization activities and disruptions to poliovirus surveillance. When the COVID-19 emergency subsides, enhanced support will be needed to resume polio eradication field activities.

## Poliovirus Vaccination

Since May 2016, after trivalent OPV (tOPV, containing types 1, 2, and 3 Sabin strains) was withdrawn from use, only bivalent OPV (bOPV; containing types 1 and 3 Sabin strains) and injectable inactivated poliovirus vaccine (IPV, containing antigens for all three serotypes) have been used in routine immunization programs worldwide. In 2018,^†^ estimated global coverage with at least 3 doses of poliovirus vaccine (Pol3) among infants aged <1 year received through routine immunization services was 89%, and with at least the recommended one full dose or two fractional doses of IPV (IPV1) was 72%. Regional, national, and subnational coverage estimates varied widely. In 2018, estimated national Pol3 coverage in Afghanistan was 73%, and IPV1 coverage was 66%; coverage in Pakistan was 75% for both Pol3 and IPV1 (*5*).

In 2018, approximately 1.2 billion bOPV, 32 million IPV, and 16 million monovalent OPV type 1 (mOPV1) doses were administered in 35 countries during 105 supplementary immunization activities (SIAs)^§^ supported by GPEI. In 2019, approximately 1 billion bOPV, 17 million IPV, and 36 million mOPV1 doses were administered in 34 countries during 90 SIAs. Since the global withdrawal of OPV2, the World Health Organization (WHO) Director-General must authorize release of monovalent OPV type 2 (mOPV2) for use in countries experiencing cVDPV2 outbreaks; in 2018, 100 million mOPV2 doses were used for outbreak response, 190 million in 2019, and 60 million in 2020 to date.

## Poliovirus Surveillance

WPV and cVDPV transmission is primarily detected by surveillance for acute flaccid paralysis (AFP) among children aged <15 years and confirmed by stool specimen testing in WHO-accredited laboratories within the Global Polio Laboratory Network. AFP surveillance performance indicators^¶^ for 40 countries during 2018–2019 have recently been reported (*6*). Among the 22 countries reporting WPV or cVDPV cases in 2018 and 2019, 11 (Afghanistan, Benin, Burkina Faso, Burma [Myanmar],** Chad, Ethiopia, Ghana, Nigeria, Pakistan, Somalia, and Zambia) met threshold criteria for the two main indicators for adequate AFP surveillance nationally during both years; five countries (Central African Republic [CAR], the Democratic Republic of the Congo [DRC], Malaysia, Papua New Guinea, and the Philippines) did not meet criteria for adequate surveillance either year; and five countries (Angola, Indonesia, Mozambique, Niger, and Togo) met criteria for both surveillance indicators in 2018, but not in 2019. Indicators vary substantially at subnational levels; national level indicators often obscure subnational underperformance (*7*). Many countries with and without recent poliovirus transmission supplement AFP surveillance with environmental surveillance (the testing of sewage for poliovirus), that allows more rapid and sensitive detection of poliovirus circulation where implemented. Persistent gaps in quality poliovirus surveillance are evident when genomic sequencing of isolates identifies polioviruses after long periods of undetected circulation. Continued strengthening of surveillance systems is necessary to confirm absence of poliovirus transmission.

## Reported Poliovirus Cases and Isolations

**Countries reporting WPV cases and isolations.** No WPV cases have been identified outside of Afghanistan, Nigeria, and Pakistan since 2015; the most recent reported onset of a WPV1 case in Nigeria was in September 2016. In 2018, 33 WPV1 cases were reported worldwide: 21 (64%) in Afghanistan and 12 (36%) in Pakistan (Figure) (Table 1).

**FIGURE:**
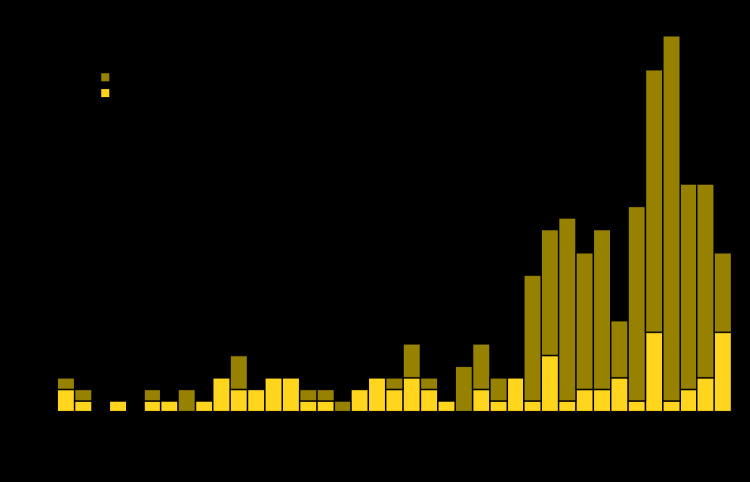
Number of cases of wild poliovirus, by country and month of onset — worldwide, January 2017–March 2020* * Data are as of June 19, 2020.

**TABLE 1:** Number of poliovirus cases, by country — worldwide, January 1, 2018–March 31, 2020*

Country	Reporting period							
	2018		2019		Jan–Mar 2019		Jan–Mar 2020	
	WPV1	cVDPV	WPV1	cVDPV	WPV1	cVDPV	WPV1	cVDPV
**Countries with endemic WPV1 transmission**								
Afghanistan	21	0	29	0	6	0	12	2
Nigeria	0	34	0	18	0	8	0	1
Pakistan	12	0	147	22	6	0	42	44
**Countries with reported cVDPV cases**								
Angola	0	0	0	130	0	0	0	2
Benin	0	0	0	8	0	0	0	1
Burkina Faso	0	0	0	1	0	0	0	4
Burma (Myanmar)^†^	0	0	0	6	0	0	0	0
Cameroon	0	0	0	0	0	0	0	3
Central African Republic	0	0	0	21	0	0	0	1
Chad	0	0	0	10	0	0	0	13
China	0	0	0	1	0	0	0	0
Côte d’Ivoire	0	0	0	0	0	0	0	5
Democratic Republic of the Congo	0	20	0	88	0	2	0	5
Ethiopia	0	0	0	13	0	0	0	14
Ghana	0	0	0	18	0	0	0	11
Indonesia	0	1	0	0	0	0	0	0
Malaysia	0	0	0	3	0	0	0	1
Mali	0	0	0	0	0	0	0	1
Mozambique	0	1	0	0	0	0	0	0
Niger	0	10	0	1	0	0	0	4
Papua New Guinea	0	26	0	0	0	0	0	0
Philippines	0	0	0	15	0	0	0	1
Somalia	0	12^§^	0	3	0	1	0	0
Togo	0	0	0	8	0	0	0	7
Zambia	0	0	0	2	0	0	0	0

**Abbreviations:** cVDPV = circulating vaccine derived poliovirus; WPV1 = wild poliovirus type 1.

* Data are as of June 19, 2020.

^†^ For this country, *MMWR* uses the U.S. State Department short-form name “Burma”; the World Health Organization uses “Myanmar.”

^§^ One patient was coinfected with type 2 and type 3 cVDPV polioviruses.

Among 176 WPV1 cases reported during 2019, 29 (16%) were reported by Afghanistan, representing a 38% increase over the 21 cases reported in 2018. Cases were reported from 20 districts, a 43% increase from the 14 districts reporting cases during 2018. Among 54 WPV1 cases detected during January–March 2020, 12 (22%) cases were detected in 11 districts of 10 provinces in Afghanistan, compared with six cases reported in six districts of three provinces during the same period in 2019. In Afghanistan, WPV1 was detected in 83 (25%) of 336 sewage samples collected from 15 of 20 (75%) sites at regular intervals in 2018 and 56 (22%) of 259 samples from 12 of 21 (57%) sites in 2019 (Table 2).

**TABLE 2:** Number of circulating wild polioviruses (WPV) and circulating vaccine derived polioviruses (cVDPV) detected through environmental surveillance — worldwide, January 1, 2018–March 31, 2020*

Country	Jan 1–Dec 31, 2018		Jan 1–Dec 31, 2019		Jan 1–Mar 31, 2019		Jan 1–Mar 31, 2020	
	No. of samples	No. (%) of isolates	No. of samples	No. (%) of isolates	No. of samples	No. (%) of isolates	No. of samples	No. (%) of isolates
**Countries with reported WPV1 cases (no. and % of isolates refer to WPV1)**								
Afghanistan	336	83 (25)	259	56 (22)	69	22 (32)	88	9 (14)
Pakistan	689	139 (20)	786	371 (47)	179	86 (47)	201	123 (61)
**Countries with reported cVDPV cases^†^ (cVDPV type) (no. and % of isolates refer to cVDPVs)**								
Afghanistan (2)	336	0 (—)	259	0 (—)	69	0 (—)	88	17 (19)
Angola (2)	106	0 (—)	106	17 (16)	24	0 (—)	13	0 (—)
Benin (2)	0	—	37	0 (—)	0	—	15	0 (—)
Burkina Faso (2)	50	0 (—)	52	0 (—)	12	0 (—)	18	0 (—)
Burma (Myanmar)^§^ (1)	59	0 (—)	12	0 (—)	9	0 (—)	6	0 (—)
Cameroon (2)	684	0 (—)	602	4 (1)	130	0 (—)	65	1 (2)
Central African Republic (2)	128	0 (—)	149	9 (6)	28	0 (—)	24	2 (8)
Chad (2)	151	0 (—)	198	10 (5)	46	0 (—)	30	3 (10)
China (2)	171	1 (1)	201	0 (—)	49	0 (—)	51	0 (—)
Cote d’Ivoire (2)	173	0 (—)	154	7 (5)	42	0 (—)	48	24 (50)
Democratic Republic of the Congo (2)	189	1 (1)	294	0 (—)	61	0 (—)	45	0 (—)
Ethiopia (2)	81	0 (—)	140	2 (1)	38	0 (—)	15	0 (—)
Ghana (2)	33	0 (—)	202	17 (9)	46	0 (—)	52	16 (31)
Indonesia (1)	117	0 (—)	174	0 (—)	45	0 (—)	31	0 (—)
Malaysia (1, 2)	0	—	60	15 (25)	10	0 (—)	177	11 (6)
Mali (2)	51	0 (—)	48	0 (—)	12	0 (—)	12	0 (—)
Mozambique (2)	90	0 (—)	76	0 (—)	15	0 (—)	15	0 (—)
Niger (2)	221	0 (—)	293	0 (—)	66	0 (—)	59	0 (—)
Nigeria (2)	166	44 (27)	211	64 (30)	483	38 (8)	347	0 (—)
Pakistan (2)	689	0 (—)	786	39 (5)	179	0 (—)	201	13 (6)
Papua New Guinea (1)	17	7 (41)	75	0 (—)	23	0 (—)	0	—
Philippines (1, 2)	87	0 (—)	212	33 (16)	30	0 (—)	87	4 (5)
Somalia (2)	422	30 (7)	92	5 (5)	32	2 (6)	25	8 (32)
Togo (2)^¶^	0	—	0	—	0	—	0	—
Zambia (2)	130	0 (—)	256	0 (—)	65	0 (—)	14	0 (—)

**Abbreviations:** cVDPV = circulating vaccine derived poliovirus; WPV1 = wild poliovirus type 1.

* Data are as of June 19, 2020.

^†^ cVDPV2 was isolated from environmental samples in Kenya (2018) and in Malaysia (2019–2020), but these isolations were not associated with cVDPV2 acute flaccid paralysis cases.

^§^ For this country, *MMWR* uses the U.S. State Department short-form name “Burma”; the World Health Organization uses “Myanmar.”

^¶^ Country does not conduct environmental surveillance.

Pakistan reported 147 (84%) of the 176 WPV1 cases in 2019, an elevenfold increase over the 12 cases reported in 2018; cases were reported in 43 districts, a sixfold increase over the six districts with confirmed cases in 2018. During January–March 2020, 42 (78%) WPV1 cases were detected in four provinces (Balochistan, Khyber Pakhtunkhwa, Punjab, and Sindh), a sixfold increase over the six cases in three provinces (Khyber Pakhtunkhwa, Punjab, and Sindh) reported during the corresponding period in 2019. In Pakistan, WPV1 was detected in 139 (20%) of 689 environmental surveillance samples from 37 of 58 (64%) sites in 2018 and 371 (47%) of 786 samples from 56 of 60 (93%) sites in 2019 (Table 2). WPV1 of Pakistan origin was detected in three environmental surveillance samples in Iran in early 2019.

**Countries reporting cVDPV cases and isolations.** During January 2018–March 2020, cVDPV transmission was confirmed in 26 countries. Five countries (Burma [Myanmar], Indonesia, Malaysia, Papua New Guinea, and the Philippines) reported four cVDPV type 1 emergences, with isolates from 39 AFP cases and 40 environmental surveillance samples. Twenty-three countries (Afghanistan, Angola, Benin, Burkina Faso, Cameroon, CAR, Chad, China, Côte d’Ivoire, DRC, Ethiopia, Ghana, Kenya, Malaysia, Mali, Mozambique, Niger, Nigeria, Pakistan, Philippines, Somalia, Togo, and Zambia) reported 49 cVDPV2 emergences, with isolates from 547 AFP cases in 21 countries and 354 environmental surveillance samples in 15 countries. Among these, the JIS-1 Nigeria emergence has spread to nine countries (*3*,*4*,*6*). Emergence of cVDPV type 3 was detected in Somalia during 2018–2019, involving isolates from seven AFP cases^††^ and 11 environmental surveillance samples.

## Discussion

WPV type 2 was certified as eradicated in 2015, and in October 2019, eradication of indigenous WPV type 3, last detected in 2012, was certified. Nigeria, the only country in the WHO African Region with indigenous WPV1 transmission after 2004, has had no evidence of circulation since September 2016; immunization coverage and surveillance in security-compromised northeast Nigeria have continued to improve. With no evidence of any WPV transmission since September 2016, the African Region meets the 3-year threshold without WPV detection required for certification and is eligible to be certified polio-free in 2020.^§§^

During January 2018–March 2020, however, transmission of both WPV1 and cVDPV2 markedly increased. Despite 4 years (2014–2017) of declines in reported WPV1 cases in Afghanistan and Pakistan, the high proportion of environmental surveillance samples with isolation of WPV1 during that time indicated persistent transmission in the historic polio reservoirs. Both countries face ongoing challenges, including vaccine refusals, polio campaign fatigue, and reaching mobile populations (*8*,*9*). In Afghanistan, antigovernment elements banned house-to-house vaccination in most southern and southeastern provinces during May–December 2018, then permitted vaccination only at designated community sites during January–April 2019 (*9*). Vaccination campaigns were banned nationally from the end of April 2019 to the end of September 2019. In Pakistan, the proportion of WPV1-positive sewage samples increased in early 2018; the number of WPV1 cases began to rise in late 2018. In 2019, the Pakistan polio program underwent a management review and is modifying its approach to address longstanding community mistrust and vaccine hesitancy issues (*8*).

The frequency and geographic extent of cVDPV2 outbreaks also increased during the reporting period, primarily because of the limited timeliness, quality, or scope of mOPV2 outbreak response SIAs and the seeding of new emergences of cVDPV2 outside mOPV2 outbreak response areas. Since 2018, cVDPV2 outbreaks have affected three of six WHO regions; most of the 23 affected countries are in Africa but also include Afghanistan and Pakistan, where WPV1 is endemic. Preparations continue for use in late 2020 of a genetically stabilized novel OPV2 (nOPV2), which has a substantially lower risk of reversion to neurovirulence and seeding new VDPV2 emergences than does Sabin mOPV2 (*10*); nOPV2 will eventually replace mOPV2 in cVDPV2 outbreak response SIAs.

In March 2020, GPEI committed to using its extensive laboratory and surveillance network and thousands of trained frontline polio workers to fully support country preparedness and response to the global COVID-19 pandemic.^¶¶^ To comply with global guidance on physical distancing during the COVID-19 pandemic, WHO and other GPEI partners recommended postponing all outbreak response SIAs until at least June 2020, and all preventive SIAs until the second half of 2020, with resumption depending upon COVID-19 control status. Although routine immunization services have been disrupted in most countries during the pandemic, GPEI is working to strengthen immunization services for preventing outbreak-prone diseases, including poliomyelitis and measles. GPEI has prioritized the continuation of AFP and environmental surveillance activities to monitor the extent of poliovirus circulation during the coming months; however, disruptions are occurring in the detection and investigation of AFP cases and in the shipping and testing of stool and sewage samples. Despite these disruptions, new areas of circulation have been identified, and preparations are underway to respond in the near future.

To address the reasons for increased WPV1 transmission since 2018 and resume field activities deferred because of response to COVID-19, it will be important for both Afghanistan and Pakistan programs to revitalize community engagement to combat polio campaign fatigue and vaccine hesitancy, strengthen the provision of basic health services, and substantially improve the management and quality of immunization activities to reach chronically missed children. In Afghanistan, continued negotiations with local antigovernment elements to resume house-to-house vaccination campaigns is crucial to reaching population immunity necessary to interrupt virus transmission. In Pakistan, implementing the 2019 management review recommendations to improve program oversight, managerial processes, and operational effectiveness is critical to strengthening SIA implementation above performance to date in all WPV1 reservoirs; identifying and mitigating the underlying challenges in underperforming districts is essential to ultimately interrupt all WPV1 transmission. In addition, defining a broad strategy to more effectively reach underserved minorities, including Pashtun populations, will be essential. Resuming preventive and outbreak response SIAs that have been paused because of the COVID-19 pandemic is critical to ensuring continued progress toward polio eradication during 2020. In the interim, GPEI and affected countries are actively planning for the safe resumption and scale-up of polio field activities when and where the COVID-19 emergency allows.

**Summary**

**What is already known about this topic?**

Wild poliovirus type 1 (WPV1) transmission continues in Afghanistan and Pakistan. Circulating vaccine-derived poliovirus (cVDPV) outbreaks occur in areas with low immunization coverage.

**What is added by this report?**

Although WPV1 incidence declined annually during 2015–2017, cases in Afghanistan and Pakistan have increased since 2018. The number and geographic spread of cVDPV type 2 (cVDPV2) outbreaks are increasing. The COVID-19 pandemic has resulted in suspension of immunization activities and disruption of poliovirus surveillance.

**What are the implications for public health practice?**

Substantial efforts to address programmatic challenges are essential to safely restore and scale-up polio field activities in 2020, including use of a stabilized type 2 oral poliovirus vaccine to prevent new cVDPV2 emergences.

## Footnotes

* https://www.who.int/news-room/feature-stories/detail/two-out-of-three-wild-poliovirus-strains-eradicated.

† 2018 is the most recent year for which data are available.

§ SIAs are mass immunization campaigns intended to interrupt poliovirus circulation by immunizing every child aged <5 years with 2 OPV doses, regardless of previous immunization status.

¶ The two main indicators of adequate AFP surveillance are 1) an annual nonpolio AFP detection rate of ≥1 case per 100,000 population aged <15 years for countries in WHO regions certified as polio-free, or ≥2 cases per 100,000 population aged <15 years in all other countries and 2) collection of adequate stool specimens (two stool specimens collected >24 hours apart, within 14 days of paralysis onset, with arrival at the laboratory in good condition [cool and without leakage or desiccation]) from ≥80% of reported AFP cases.

** For this country, *MMWR* uses the U.S. State Department short-form name “Burma”; WHO uses “Myanmar.”

†† One AFP case detected in Somalia was coinfected with cVDPV type 2 and type 3.

§§ https://www.who.int/bulletin/volumes/82/1/24-30.pdf?ua=1.

¶¶ http://polioeradication.org/news-post/call-to-action-to-support-covid-19-response/.
